# Association of AQP5 gene variants and mRNA expression with caries severity in a dental practice cohort: a randomized trial

**DOI:** 10.1038/s41598-026-59514-7

**Published:** 2026-06-30

**Authors:** Janusch Bitter, Daria Pakosch-Nowak, Michael Adamzik, Dominik Ziehe, Jennifer Orlowski, Bjoern Koos, Martin Kunkel, Katharina Rump, Markus Baumann

**Affiliations:** 1 Zahnarztpraxis Dr. Baumann Sprockhövel, Sprockhövel, Germany; 2 Department of Anesthesiology, Intensive Care Medicine and Pain Therapy, Knappschaft Kliniken University Hospital Bochum, Ruhr University Bochum, Bochum, Germany; 3 Department of Oral and Maxillofacial Surgery, Knappschaft Kliniken University Hospital Bochum, Ruhr University Bochum, Bochum, Germany

**Keywords:** AQP5, Genetic variants, mRNA, Saliva, Caries, Biomarkers, Diseases, Genetics, Medical research

## Abstract

Aquaporin 5 (AQP5) is crucial for salivary secretion, composition and enamel mineralization. Genetic variations in AQP5 may influence susceptibility to dental diseases such as caries. This study investigated the association between AQP5 single nucleotide polymorphisms (SNPs), AQP5 mRNA expression, and caries severity in a dental practice cohort. A total of 246 patients were enrolled. Caries experience was assessed using the decayed, missing, and filled (DMF) index, and salivary AQP5 mRNA expression was quantified by RT-qPCR. Genotyping included rs2878771, rs296763, rs3736309, and rs3759129. Statistical analyses comprised Chi-square testing, ROC analysis with Youden Index determination, and binary logistic regression adjusted for age and sex. The A allele of rs3736309 was associated with an increased risk of severe caries, particularly in patients over 60 years of age. AQP5 mRNA expression was higher in individuals with severe caries and in carriers of the C allele of rs2878771. ROC analysis identified an AQP5 expression cut-off (0.11274) that discriminated between severe and non-severe caries (AUC = 0.578, *p* = 0.048). Logistic regression confirmed AQP5 expression as an independent predictor of severe caries (*p* = 0.005). AQP5 expression and genetic variation appear to contribute to caries susceptibility in an age-dependent manner with moderate effects. The intronic variant rs3736309 was associated with caries severity. The biological mechanisms underlying this association remain unclear and require functional investigation. These findings support a potential role of AQP5 as an exploratory biomarker candidate for caries risk, particularly in elderly individuals.

*Trial registration*: German Clinical Trial Registry No. DRKS00032425, date of registration: 2023-08-16.

## Introduction

Dental caries remains one of the most common chronic diseases worldwide and results from a multifactorial interplay between microbial, environmental, and host-related factors^1^. Among host determinants, saliva plays a pivotal role in maintaining oral homeostasis through its buffering capacity, antimicrobial activity, and mineral content^2^. The composition and secretion of saliva are tightly regulated by aquaporins (AQPs), a family of water channel proteins that facilitate rapid transmembrane water transport^3^.

AQP5, one of the major aquaporins expressed in salivary gland acinar cells, is crucial for saliva secretion and fluid homeostasis in the oral cavity^4^. Altered localization or reduced expression of AQP5 has been associated with salivary gland dysfunction in Sjögren’s syndrome, diabetes, and aging, supporting its role in maintaining normal salivary flow and function^5,6^. In our previous work, we demonstrated that AQP5 mRNA shows the highest abundance among all aquaporins detectable in human saliva, underscoring its potential relevance for oral health and disease^7^.

Several studies have explored genetic variation in AQP5 in relation to caries susceptibility^8^. Multiple single nucleotide polymorphisms (SNPs) within the AQP5 gene have been associated with altered caries experience; however, most of these studies were limited to genotypic correlations and lacked functional validation^9^. Only one prior study directly linked AQP5 expression to caries risk, reporting that higher AQP5 expression was associated with lower caries experience, suggesting a potential protective role^10^. Furthermore, AQP5 mRNA has been detected in human saliva in two small studies, indicating its feasibility as a noninvasive biomarker, though these findings were limited by sample size and methodological constraints^7,10^. Overall, the current evidence on AQP5 and caries remains inconsistent and of limited quality, warranting further mechanistic investigation^11^.

In the present study, we aimed to address these gaps by combining gene expression and genetic association analyses of AQP5 in human saliva. Specifically, we investigated whether salivary AQP5 mRNA expression correlates with caries experience and whether genetic variants in AQP5, particularly influence its expression and caries susceptibility.

## Methods

### Study design and cohort

The OKAPI study (German Clinical Trial Registry No. DRKS00032425; registered on 2023-08-16) prospectively included patients who fulfilled the predefined inclusion criteria^12,13^. This was an exploratory observational cross-sectional study. Patients were consecutively recruited from a dental practice cohort. No intervention, randomization, treatment allocation, or comparison of therapeutic strategies was performed. Ethical approval was granted by the Ethics Committee of the Medical Faculty of Ruhr University Bochum (reference no. 23-7821-BR; approved on 07 June 2023) as well as by the Ethics Committee of the Westphalia-Lippe Medical Association (reference no. 2023-416-b-S; approved on 26 July 2023). All relevant study documents—including the study protocol, site-specific informed consent forms, participant information and recruitment materials, as well as any amendments—were reviewed and approved by the respective ethics committees^12,13^.

The study was conducted in accordance with the revised Declaration of Helsinki, Good Clinical Practice guidelines, and applicable local regulatory requirements. Patients or the public were not involved in study design. Participants were recruited over a 17-month period in a dental practice after providing written informed consent. Eligible participants were adult dental patients aged 18 to 75 years. Exclusion criteria comprised hereditary structural abnormalities of dental hard tissues (e.g., amelogenesis imperfecta, dentinogenesis imperfecta, odontogenesis imperfecta), dementia and/or psychotic disorders, inability to provide informed consent, and insufficient proficiency in the German language to adequately understand the study procedures and requirements^12,13^.

### Clinical data

When assessing the prevalence of caries, a patient is considered to have caries if at least one of the following criteria is met^12,13^:

- Active dental caries on one or more teeth
- Missing tooth/teeth (extraction due to caries)
- Presence of one or more restored teeth by means of a filling or prosthetic restoration (e.g. dental crown)

In this study, we defined the severity of caries as follows:

- severe caries: 15 or more teeth meeting the above-mentioned criteria
- Medium caries: six to 14 affected teeth
- Mild caries: five or fewer affected teeth

In addition to recording the caries status and collecting biosamples, clinical data and risk profiles were collected via a questionnaire. This survey included the following measurement variables^12,13^:

- *Age*
- Gender (m/f/d)
- Pre-existing medical conditions:
  - arterial hypertension, diabetes mellitus
  - Cardiovascular diseases (including CHD, post-myocardial infarction, PAD, etc.)
  - Nicotine abuse
  - Current or previous malignant tumor disease: localization, TNM stage, radio and/or chemotherapy
  - Long-term medication (e.g. antiresorptive or immunosuppressive medication)
- Periodontitis

### Collection of samples

As part of the dental treatment in the dental practice, patients included in the study provided an oral mucosal swab and 2 ml saliva samples^12,13^. Mucosal swabs were utilized for gDNA isolation utilizing my-Budget DNA Mini Kit (BioBudget, Krefeld, Germany). DNA was quantified using NanoDrop™ One (Thermo Fisher Scientific, Waltham, MA USA). The saliva samples were collected into Saliva RNA Collection and Preservation Devices (Norgen Biotek). These samples were then used to isolate RNA using the Total RNA Purification Kit (Norgen Biotek). After isolation, the samples were stored at − 80 °C^12,13^. Prior cDNA synthesis RNA quantity and quality was assessed using NanoDrop™ One. For quality assessment the A260/A280 and A260/A230 ratios were utilized.

### RNA quantification

For RNA quantification, a two step RT qPCR method was utilized, which consisted of cDNA synthesis and qPCR reaction. For cDNA synthesis, 1 µg of RNA was used with the High-Capacity cDNA Reverse Transcription Kit (Thermo Fisher Scientific, Wilmington, USA)^12,13^. This method uses RT Random Primers for cDNA synthesis. After the RNA was transcribed into cDNA, it was analyzed using qPCR for the expression analysis of candidate genes. Specific primers for AQP5 were used: AQP5_RT_2_SETCGGTTCAGCCCCGCTCACT and AQP5_RT_2_ASGCCACACGCTCACTCAGGCT -3′. The expression was quantified using the ΔCt method, with β-Actin (ACTB) serving as the reference gene, as described previously^14,15^.

### SNP TaqMan assay for genotyping

To analyze the SNPs rs2878771, rs3736309, rs296763, and rs3759129 in samples obtained from 246 patients, TaqMan SNP genotyping assays (Thermo Fisher Scientific, Darmstadt, Germany) were employed in a quantitative PCR setup (CFX Connect Real-Time PCR System, Bio-Rad Laboratories, Hercules, CA, USA) using TaqMan Genotyping Master Mix (Thermo Fisher Scientific, Darmstadt, Germany). The selected SNPs were identified based on information retrieved from the NCBI database.

For each reaction, a total volume of 25 µL was prepared, consisting of 24 µL of SNP reaction mix (12.5 µL Master Mix, 1.25 µL TaqMan SNP assay primer, and 10.25 µL nuclease-free water) and 1 µL of genomic DNA (10 ng/µL), which was dispensed into an optical reaction plate. Amplification was carried out with an initial denaturation step at 95 °C for 10 min, followed by 40 cycles of denaturation at 95 °C for 15 s and annealing/extension at 60 °C for 1 min. Genotype determination was performed based on fluorescence signals measured for each sample at the VIC and FAM wavelengths.

### Statistical analysis

No formal a priori sample size calculation was performed because this study was designed as an exploratory observational investigation. The sample size was determined by the number of eligible participants who were recruited during the study period and who provided complete clinical and biological samples for analysis.

Patient characteristics are presented as percentages for categorical variables and as either means with standard deviations (SD) or medians with interquartile ranges (25th–75th percentiles), depending on data distribution. Comparisons of categorical variables were performed using McNemar’s test or Fisher’s exact test, as appropriate. Continuous variables were analyzed using either the Student’s t-test or the Mann–Whitney U test after assessment of normality with the Shapiro–Wilk test^12,13^.

Thresholds for AQP5 mRNA expression to distinguish between patients with and without caries were identified using receiver operating characteristic (ROC) curve analysis. The optimal cut-off point was determined using the Youden index and subsequently incorporated into a binary logistic regression model^12,13^.

For the genetic analyses, genotype distributions were first evaluated for compliance with Hardy–Weinberg equilibrium (HWE). In addition, genotype frequencies were compared with reference data from the NCBI SNP database using the chi-square test. A binary logistic regression analysis was then conducted to investigate the association between age, expression cut-off values, and the likelihood of caries. This model included age, disease status (yes/no), and whether the defined expression threshold was exceeded. Based on this, predicted probabilities of disease were calculated across different age values within both categories^16,17^.

A *p*-value < 0.05 was considered statistically significant. Unless otherwise specified, data are presented as mean ± standard deviation (SD). Statistical analyses were performed using SPSS (version 28, IBM, Chicago, IL, USA), and graphical visualizations were created with GraphPad Prism 9 (GraphPad Software, San Diego, CA, USA).

## Results

### Baseline characteristics

We collected patients in the dental practice and our cohort consisted of 246 patients of a dental practice. Most of the patients (57.3%) were females and the median age was 46 years (Table 1). Only 23.6% of the cohort had no or mild caries. The most common comorbidity was arterial hypertonia. Genotypes are represented in total amounts. Percentages display percentage distribution in the study cohort and in NCBI database (second bracket, here referred as reference cohort) (Table 1).

**Table 1:** Baseline characteristics of the study cohort.

	N = 246
Gender male	105 (42.7%)
Age median (IQR)	46 (35–56)
Caries	
No to mild caries	89 36.2%)
Medium caries	59 (24.0%)
Severe caries	98 (39,8%)
Comorbidities	
Arterial hypertonia (yes)	40 (16.3%)
Heart disease	13 (5.3%)
Smoking (yes)	41 (16.7%)
Smoking in years	4.025 ± 10.8
Diabetes	3 (1.2%)
Cancer	2 (0.8%)
Genotypes	
rs2878771	GG: 157 (65.4%) (78.1%)
	GC: 70 (29.3%) (19.5%)
	CC: 13 (5.4%) (2.3%)
rs296763	GG: 165 (67,1%) 56.7
	CG: 70 (28.5%) 37.2
	CC: 11 (4.5%) 3.6%
rs3736309	AA: 160 (65%) (71.7%)
	AG: 62 (25.2%) (25.9%)
	GG: 6 (2.4%) (2.3%)
rs3759129	AA: 213 (86.6%) (65.4%)
	AC: 19 (7.7%) (30.9%)
	CC: 3 (1.2%) (3.6%)

### Genotype-dependent distribution: study cohort versus reference database

**Fig. 1:**
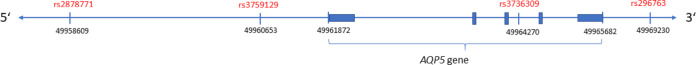
Localization of *AQP5* polymorphisms analyzed in this study.Schematic representation of the human AQP5 gene located on chromosome 12q13.12,illustrating the genomic positions of the investigated single nucleotide polymorphisms (SNPs)rs2878771, rs296763, rs3736309, and rs3759129. The AQP5 gene spans approximately 3.8 kbon chromosome 12 (GRCh38: chr12:49,961,811–49,965,682) and consists of fi ve exonsseparated by four introns. The positions of the SNPs are indicated relative to the genestructure and transcriptional orientation. These variants were selected to capture geneticvariation across the AQP5 locus and have previously been included in association studiesinvestigating oral and systemic disease phenotypes.

The genotype distribution of rs2878771, rs296763 and rs3736309 did not differ between the reference cohort (NCBI database) and the study cohort (Chi^2^, *p* > 0.05, Table 1). However the presence of C-allele was significant lower in the study cohort compared to reference cohort (NCBI database) (Chi^2^, *p* < 0.0001, Table 1) and compared to another study cohort of our clinic^16^ (Chi2, *p* < 0.0001, (AA: 136; AC/CC: 53). The localization of the analyzed Polymorphims in the *AQP5* gene are shown in Figure 1. Two SNPs are located in the 5’UTR region, one in intron 3 and 1 SNP in the 3’UTR region (Fig. 1). 

### Genotype-dependent AQP5 mRNA expression

**Fig. 2:**
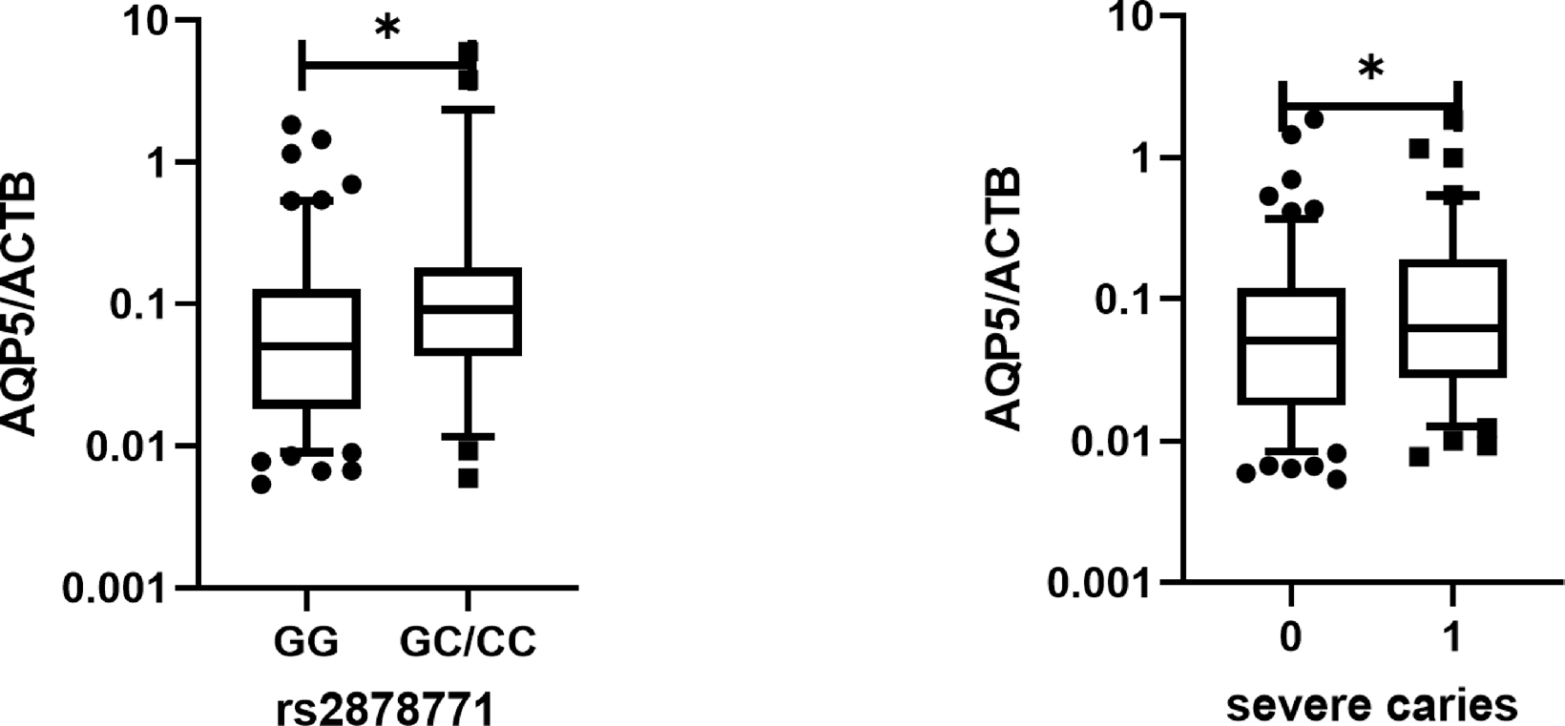
Salivary AQP5 mRNA expression according to rs278771 genotype and caries severity. (**A**) Boxplots showing AQP5 mRNA expression stratified by rs278771 genotype, with higher expression levels in carriers of the C allele (GC/CC; n = 82) compared with individuals with the GG genotype (n = 135; *p* = 0.0125). (**B**) Boxplots showing AQP5 mRNA expression according to caries severity, with higher expression observed in patients with severe caries compared with those without severe caries (*p* = 0.0389). The GG group comprised 122 individuals, and the GC/CC group comprised 54 individuals. AQP5 mRNA expression values are shown on a log₂ scale. Boxplots display the median, interquartile range, and the 5th to 85th percentiles.

In rs296763, rs3736309 and rs3759129 the AQP5 expression did not differ between genotypes (*p* > 0.05). AQP5 mRNA expression was higher in individuals carrying the C allele of rs278771 than in those with the GG genotype (*p* = 0.0125, Fig. 2a). In addition, patients with severe caries showed higher AQP5 mRNA compared to other patients (*p* = 0.0389, Fig. 2b).

### ROC analysis and Youden index for AQP5 in predicting caries

Receiver operating characteristic (ROC) analysis revealed that AQP5 expression discriminated between severe caries and non-severe caries. The optimal cut-off value determined by the Youden Index was 0.11274, with a sensitivity of 41.7% and a specificity of 74.3% (AUC: 0.578; *p* = 0.048; 95% CI: 0.501 to 0.656).

### Correlation of AQP5 genotypes with caries severity

Cross-tabulation analyses were conducted to evaluate the relationship between the investigated SNPs and the DMF status. The polymorphism rs3736309 showed a significant association with DMF status in the subgroup of older patients, suggesting a possible age-dependent genetic effect (*p* = 0.01, Table 2).

**Table 2:** Cross-tabulation analyses between genotypes and DMF status depending on age.

Polymorphism	All patients						Patients above 60 years					
	Genotype	DMF status				*p*-value	Genotype	DMF status				*p*-value
		1	2	3	4			1	2	3	4	
rs2878771	GG	41	21	38	57	0.472	GG	0	2	4	21	0.637
	GC	16	9	15	30		GC	0	1	1	9	
	CC	0	2	5	6		CC	0	1	0	2	
rs296763	GG	35	22	43	65	0.366	GG	0	3	4	23	0.262
	CG	18	7	16	29		CG	0	0	2	9	
	CC	5	2	0	4		CC	0	1	0	1	
rs3736309	AA	37	21	40	70	0.102	AA	0	2	2	26	0.010*
	AG	18	7	16	21		AG	0	1	3	7	
	GG	2	3	0	1		GG	0	1	0	0	
AQP5 (-1364) rs3759129	AA	52	32	52	83	0.480	AA	0	4	5	26	0.740
	AC	4	0	6	9		AC	0	0	0	5	
	CC	0	0	1	2		CC	0	0	0	1	

Significant values are in italic.

### Binary logistic regression analyses

In the final analysis, a binary logistic regression was conducted to evaluate the impact of the AQP5 expression cut-off and the different AQP5 genotypes on the severity of dental caries. In the overall cohort, the AQP5 cut-off exhibited the strongest association with severe caries, remaining significant after adjustment for age and sex (*p* = 0.005, Table 3). In the subgroup of older patients aged over 60 years, a substantially higher proportion of individuals presented with severe caries (79.6%) compared to the total study population (39.3%). Moreover, in this older subgroup, the AQP5 SNP rs3736309 showed a strong and statistically significant association with caries severity (*p* = 0.018, Table 4). These findings indicate that the occurrence and severity of dental caries are strongly age-dependent, and suggest that both AQP5 expression and genetic variation in AQP5 may contribute to caries susceptibility, particularly in the elderly.

**Table 3:** Binary logistic regression analysis of factors associated with caries in all study participants.

	p-value	Hazard ratio	95.0% confidence intervall	
			Lower	Upper
rs3736309 AA	0.234	0.629	0.293	1.350
AQP5 cutoff	0.005	3.070	1.394	6.759
gender	0.538	1.255	0.609	2.586
Age per year	> 0.0001	1.091	1.056	1.128
Smoking (yes)	0.810	0.888	0.336	2.343

The table presents odds ratios (ORs) with 95% confidence intervals (CIs) and p values for the association between caries status (dependent variable) and the included covariates.

**Table 4:** Binary logistic regression analysis of factors associated with caries in participants aged > 60 years.

	*p*-value	Hazard ratio	95.0% confidence intervall	
			Lower	Upper
rs3736309 AA	0.018	0.038	0.003	0.575
AQP5 cutoff	0.146	7.387	0.499	109.388
gender	0.428	0.410	0.045	3.715
Age per year	0.311	1.174	0.861	1.602
Smoking (yes)	0.533	2.049	0.215	19.533

The table presents odds ratios (ORs) with 95% confidence intervals (CIs) and p values for the association between caries status (dependent variable) and the included covariates in this age subgroup.

## Discussion

In this study, we investigated the association between AQP5 gene variants, AQP5 mRNA expression, and the severity of dental caries in a cohort of adult patients from a dental practice. To our knowledge, this is one of the first studies to link AQP5 expression and genotype distribution with caries status in a clinical cohort. Our findings suggest that both AQP5 expression levels and genetic variation in AQP5 may influence caries susceptibility. AQP5 can therefore be referred as an exploratory biomarker candidate. Based on our findings we cannot call AQP5 a clinically applicable diagnostic marker, hence our study lacks external validation.

The genotype distribution of the analyzed SNPs (rs3759129, rs296763, and rs3736309) did not differ significantly between our study population and reference data from the NCBI database. However, the frequency of the C allele of the rs278771 was markedly lower in our cohort compared to both the reference data and a previously analyzed cohort from our clinic^16,18^. This difference could reflect population-specific genetic variability or selection bias related to the clinical recruitment setting. Despite the absence of overall genotype differences, the C allele of rs2878771 was associated with increased AQP5 expression, suggesting a potential functional role in the transcriptional regulation of AQP5. As rs2878771 is located within an intronic region of the lncRNA AQP5-AS1, the variant may exert regulatory effects on AQP5 expression, potentially through modulation of transcriptional activity, RNA processing, or local chromatin architecture rather than through direct alteration of protein structure.

At the expression level, patients with severe caries showed significantly higher AQP5 mRNA compared to those with mild or no caries, supporting a role of AQP5 in caries pathophysiology. AQP5 is a key water channel in salivary glands that regulates saliva secretion and composition^7^. Reduced or dysregulated AQP5 function has been linked to xerostomia, altered salivary flow, and changes in enamel remineralization, which are critical determinants of caries susceptibility^6,10,19,20^. One possible explanation is that increased AQP5 expression reflects adaptive changes in the oral environment; however, the present study was not designed to investigate underlying mechanisms.

Although statistically significant, the discriminatory performance of AQP5 expression was limited (AUC = 0.578), indicating that AQP5 expression alone is unlikely to provide clinically useful diagnostic accuracy. The observed association may nevertheless be biologically informative and could contribute to future multivariable prediction models. The Youden Index-derived cut-off (0.11274) may serve as a reference point for future studies exploring molecular thresholds of AQP5-related susceptibility. Our data suggest that the relevance of an AQP5 expression–based cut-off decreases with increasing age. Our data cannot pinpoint out functional effects of AQP5. However we could speculate that as AQP5 expression is mechanistically linked to salivary water secretion and thereby to caries susceptibility, age-related structural and functional alterations of the salivary glands could attenuate the functional impact of AQP5 expression in older individuals. The apparent age-dependent differences observed in our cohort may reflect age-related changes in salivary gland physiology. However, no functional measurements of salivary gland activity were performed, and the underlying mechanisms remain speculative.

Interestingly, while the predictive value of AQP5 expression appears to diminish with age, the influence of AQP5-related single nucleotide polymorphisms seems to increase. One possible explanation is that genetic effects become more apparent in older individuals; however, this hypothesis requires confirmation in larger age-stratified cohorts.

The A allele of rs3736309 was associated with an increased risk of severe caries, whereas the G allele was less frequent among affected individuals. This contrasts with findings in dental fluorosis, where the G allele was more prevalent in affected patients, suggesting phenotype-specific effects of this SNP^21^. As rs3736309 is located in a non-coding region, its functional consequences remain unknown. Future studies are needed to determine whether this variant affects AQP5 expression, splicing, or other regulatory mechanisms.

Several limitations of this study should be acknowledged. First, participants were recruited from a single dental practice, which may introduce selection bias and limit the generalizability of the findings to broader populations. Second, the cross-sectional design precludes causal inference, and longitudinal studies are required to determine whether AQP5 expression or genetic variation can predict future caries development. Third, dental caries is a highly multifactorial disease. Although relevant clinical information was collected, important determinants such as dietary habits, oral hygiene practices, fluoride exposure, salivary flow rate, medication use, socioeconomic factors, and oral microbiome composition were not comprehensively assessed and may have influenced the observed associations. Fourth, age-stratified analyses, particularly in individuals older than 60 years, were based on a relatively small subgroup and should therefore be interpreted with caution because of limited statistical power and the increased risk of unstable estimates. Fifth, no independent validation cohort was available, and the observed discriminatory performance of AQP5 expression was modest (AUC = 0.578), limiting its immediate clinical applicability as a biomarker. Finally, only salivary AQP5 mRNA expression was assessed, whereas protein expression, salivary flow measurements, and functional analyses were not performed. Consequently, the biological mechanisms linking AQP5 genetic variation, gene expression, and caries susceptibility remain unclear. Therefore, the present findings should be considered exploratory and require confirmation in larger, independent, and longitudinally characterized cohorts. Despite these limitations, the present study provides evidence for an association between AQP5-related genetic and transcriptional variation and caries severity. Given the exploratory nature of the study, the modest discriminatory performance of AQP5 expression, and the lack of external validation, these findings should be interpreted cautiously and require confirmation in larger independent cohorts.

## Data Availability

Data is available from the corresponding author on reasonable request.
